# Clinical Outcomes of Once-Weekly Hypofractionated Intensity-Modulated Radiation Therapy with Concurrent α-Sulfoquinovosyl-Acylpropanediol for Modified Adams Stage 4 Canine Intranasal Tumors: A Retrospective Case Series

**DOI:** 10.3390/vetsci13060601

**Published:** 2026-06-20

**Authors:** Akihiro Ohnishi, Yuko Mizutani, Saki Kageyama, Shinya Mizutani, Taketoshi Asanuma

**Affiliations:** 1 Department of Veterinary Science, Okayama University of Science, 1-3 Ikoinooka, Imabari 794-8555, Ehime, Japan; a-oonishi@ous.ac.jp (A.O.);; 2 Graduate School of Veterinary Science, Okayama University of Science, 1-3 Ikoinooka, Imabari 794-8555, Ehime, Japan

**Keywords:** canine intranasal tumor, hypofractionated radiotherapy, intensity-modulated radiation therapy, SQAP, computed tomography, volumetric assessment

## Abstract

We described tumor response and survival in dogs with modified Adams stage 4 intranasal tumors treated with once-weekly hypofractionated radiation therapy (RT) combined with the radiosensitizer α-sulfoquinovosyl-acylpropanediol (SQAP), and compared linear and volumetric response assessments. Twenty dogs treated with intensity-modulated RT (8 Gy per fraction, once weekly) and concurrent SQAP were included in this retrospective case series. Tumor response was assessed using RECIST-like linear measurements and volumetric analysis on contrast-enhanced computed tomography. Overall survival (OS) was estimated using Kaplan–Meier analysis. Of the 20 dogs, 4 were classified as stage 4a and 16 as stage 4b. The best RECIST-like responses were complete response (CR) in 5 dogs, partial response (PR) in 12, and stable disease (SD) in 4. Volumetric responses were CR in 5 dogs, PR in 11, and SD in 5. No cases demonstrated progressive disease as the best response. The median OS for all dogs was 342 days (95% confidence interval [CI], 206–419 days). Censoring one non-tumor-related death yielded a median OS of 356 days (95% CI, 231–419 days). Exploratory analysis revealed median OS of 393 and 297 days for stage 4a and 4b dogs, respectively. Volumetric assessment appeared more sensitive for detecting tumor regrowth in selected cases. Dermatologic adverse events were limited to alopecia within the radiation field, and no complete vision loss was observed. Seizure activity was documented in eight dogs. In conclusion, once-weekly hypofractionated intensity-modulated RT combined with concurrent SQAP was associated with clinically meaningful survival outcomes in dogs with advanced intranasal tumors. However, because no radiotherapy-alone control group was available, the independent contribution of SQAP to these outcomes could not be determined.

## Simple Summary

Advanced canine intranasal tumors with cribriform plate destruction or intracranial extension are generally associated with a poor prognosis. Conventional radiation therapy protocols often require repeated anesthesia and frequent hospital visits, which may be challenging for elderly dogs and their owners. In this retrospective case series, we evaluated clinical outcomes in 20 dogs with modified Adams stage 4 intranasal tumors treated with once-weekly hypofractionated intensity-modulated radiation therapy (IMRT) combined with the radiosensitizer α-sulfoquinovosyl-acylpropanediol (SQAP). Median overall survival exceeded 11 months, and treatment-related adverse events were generally manageable. We also compared conventional linear tumor measurements with volumetric assessments using serial computed tomography and found that volumetric analysis may provide additional information regarding tumor regrowth. These findings suggest that once-weekly hypofractionated IMRT may represent a practical treatment option for selected dogs with advanced intranasal tumors while reducing treatment burden.

## 1. Introduction

Canine intranasal tumors represent a clinically challenging disease in veterinary oncology. For such tumors, particularly for non-resectable cases, radiation therapy (RT) is widely regarded as the principal treatment modality. Multiple studies have demonstrated its capacity to provide meaningful tumor control and survival benefits [1,2,3]. However, the prognosis remains guarded in advanced-stage disease, especially in cases classified as modified Adams stage 4, in which cribriform plate destruction and intracranial extension are frequently observed [4,5]. Modified Adams stage 4 disease is generally regarded as an advanced clinical stage associated with poor prognosis. Furthermore, reports specifically evaluating aggressive treatment strategies in dogs with stage 4 intranasal tumors remain limited, particularly in the setting of hypofractionated radiation protocols.

Conventional definitive-intent RT protocols typically use finely fractionated schedules that are delivered over multiple weeks. Although such approaches are associated with improved local control, they require repeated general anesthesia and frequent hospital visits. In veterinary patients, anesthesia burden represents a uniquely significant limitation compared with that in human oncology, particularly in geriatric populations and individuals with concurrent systemic disease. In addition, owner-related logistical and psychological factors often influence treatment feasibility and compliance.

Hypofractionated RT protocols, including once-weekly schedules, have emerged as practical alternatives, primarily within palliative or coarse fractionation frameworks [6,7]. These regimens substantially reduce the frequency of anesthesia and duration of treatment, thereby alleviating the burden on patients and owners. Nevertheless, concerns persist regarding the durability of tumor control and overall treatment efficacy when compared with conventional fractionation strategies.

The use of radiosensitizers has been proposed as a potential strategy to enhance RT efficacy without increasing the total radiation dose or treatment intensity. α-sulfoquinovosyl-acylpropanediol (SQAP) is a novel radiosensitizer that can inhibit DNA repair pathways and enhance radiation-induced cytotoxicity in experimental models [8,9]. SQAP has also been shown to modulate the tumor microenvironment, including improving tumor oxygenation, which may contribute to an enhanced radiation response [10]. Despite the increasing clinical interest, evidence regarding its performance in combination with hypofractionated RT protocols remains limited, particularly in advanced-stage canine intranasal tumors. Accordingly, we focused on evaluating the clinical outcomes of dogs with advanced intranasal tumors treated with once-weekly hypofractionated RT combined with SQAP.

Another unresolved issue in veterinary radiation oncology is optimal response assessment methodology. Although linear measurement-based criteria, such as RECIST-derived approaches, remain widely utilized, volumetric evaluation techniques may provide superior sensitivity for detecting subtle tumor progression or regrowth [11]. Comparative analyses of these assessment strategies for canine nasal tumors are scarce, particularly in the context of hypofractionated RT and radiosensitizer use.

To address these gaps, we aimed to describe the tumor response and survival outcomes in dogs with modified Adams stage 4 intranasal tumors treated with once-weekly hypofractionated RT combined with SQAP, characterize post-treatment tumor dynamics using serial computed tomography (CT), and compare linear (RECIST-like) and volumetric response assessment methods.

## 2. Materials and Methods

Study Design and Case Selection

We conducted a retrospective case series to evaluate the clinical outcomes and imaging-based tumor responses in dogs with advanced intranasal tumors treated with hypofractionated radiation therapy combined with a radiosensitizer. Medical records from a university veterinary teaching hospital were reviewed. Patients were eligible for inclusion if they (1) had a histologically diagnosed intranasal or sinonasal tumor, (2) were classified as modified Adams stage 4 at the time of radiation therapy, (3) were treated using a once-weekly hypofractionated radiation protocol, (4) received SQAP as a radiosensitizer, and (5) had survival and follow-up data available.

No restrictions were applied regarding tumor histopathology. Patients who received concurrent therapies, including toceranib phosphate or hyperthermia, were not excluded. Re-irradiation cases were included if treatment records and outcome data were available.

Staging and Substage

Tumors were staged according to the modified Adams staging system. Substage classification (4a vs. 4b) was based on imaging findings. Stage 4b was assigned when imaging demonstrated intracranial extension or loss of the normal anatomical boundary between the tumor and cranial cavity structures. Patients without imaging evidence of intracranial involvement were classified as stage 4a disease.

RT Protocol

RT planning was based on contrast-enhanced CT acquired prior to treatment initiation. CT was conducted with patients under general anesthesia and positioned in sternal recumbency using a customized immobilization system. All dogs were immobilized using a vacuum cushion, thermoplastic mask, and individualized dental bite block to improve interfractional reproducibility and minimize rotational and positional variability.

CT datasets were acquired with a slice thickness of 2 mm. Contrast enhancement was performed routinely, and all included patients underwent contrast-enhanced CT to facilitate accurate gross tumor delineation and assessment of adjacent structures.

Treatment was performed using a dedicated RT planning system (Monaco, Elekta AB, Stockholm, Sweden). Target volumes were defined according to established RT principles. The gross tumor volume was contoured based on visible tumor extent identified on contrast-enhanced CT images. The clinical target volume was defined by considering potential microscopic extension, anatomical barriers, and tumor characteristics. The planning target volume margin was determined individually for each case, with modifications based on the proximity of organs at risk (OARs) and relevant histopathological considerations. In general, an isotropic expansion of approximately 2 mm was applied as a practical guideline while allowing clinician-directed adjustments to balance tumor coverage and normal tissue sparing.

The bilateral globes and brain were contoured as OARs in all cases. Dose distribution was optimized to prioritize ocular and intracranial protection while maintaining adequate target coverage. Dose constraints were applied in accordance with institutional practice and published recommendations for veterinary RT. Treatment plans were generated using a step-and-shoot intensity-modulated radiation therapy (IMRT) technique with 6 MV photon beams.

The standard treatment protocol consisted of five once-weekly fractions. One dog received a sixth fraction because olfactory neuroblastoma was initially suspected based on histopathological findings, raising concern for a highly locally invasive tumor. Additional tissue sampling was performed during treatment for further diagnostic evaluation, and the final diagnosis of adenocarcinoma was established after completion of radiotherapy. Therefore, the sixth fraction reflected a case-specific clinical decision rather than a planned treatment modification based on treatment response.

SQAP was administered intravenously at a dose of 4 mg/kg before each irradiation session. In accordance with the institutional protocol, the timing of administration was adjusted to allow radiation delivery approximately 15–30 min after SQAP administration. Patient positioning was verified before each fraction using portal imaging. Positional corrections were applied when necessary to achieve acceptable alignment with the treatment plan. All treatments were performed using a linear accelerator.

Concurrent and Adjunctive Therapies

Adjunctive therapies were administered on a case-by-case basis according to clinician judgement, tumor characteristics, patient condition, and owner preference. Toceranib phosphate was administered to a subset of dogs after completing RT. The initial dose was 3.0–3.25 mg/kg, administered every other day as the standard starting regimen. Dose and dosing intervals were adjusted according to the severity of adverse events observed during treatment. If adverse effects prevented continuous administration for at least 2 weeks, the patient was excluded from the combination therapy group. Hyperthermic treatment was performed in selected cases using a radiofrequency thermal therapy system (AMTC 300 B; AdMeTech Co., Ltd., Ehime, Japan). Under CT guidance, a heating probe was inserted directly into the tumor mass. The insertion depth was determined based on the CT images to ensure that the heating element was positioned at least 1 cm inside the tumor margin. For tumors located within the nasal cavity, the probe was inserted through the nostril, whereas tumors involving the frontal sinus were approached percutaneously through the skin. Tumor heating was performed at 65 °C for 8 min, followed by 80 °C for 15 s to promote coagulation and minimize bleeding. To reduce potential inflammatory reactions associated with treatment, prednisolone was administered at an anti-inflammatory dose of 0.5 mg/kg once daily when the radiation field was adjacent to the brain or ocular structures.

Re-irradiation was proposed when follow-up CT examinations demonstrated local tumor regrowth after completion of the initial radiotherapy course. The decision to proceed with re-irradiation and the number of additional fractions administered were determined individually based on clinical condition, owner preference, and financial considerations. Details of re-irradiation were individualized and are therefore reported descriptively. Survival outcomes included the entire clinical course after initial RT, including periods after re-irradiation when applicable. Use of SQAP during re-irradiation was determined on a case-by-case basis.

Response Evaluation

The tumor response to radiation therapy was assessed using both linear (RECIST-like) and volumetric measurement approaches based on follow-up contrast-enhanced CT imaging. For the RECIST-like method, which was adopted for the linear assessment, the longest tumor dimensions were measured on multiplanar reconstructed images according to the Veterinary Cooperative Oncology Group (VCOG) response evaluation criteria for solid tumors in dogs [11]. Response categories were defined as follows: complete response (CR), disappearance of the measurable lesion; partial response (PR), ≥30% reduction in the summed linear dimensions relative to baseline; progressive disease (PD), ≥20% increase relative to baseline; and stable disease (SD), changes not meeting PR or PD criteria.

Volumetric assessment was performed using Horos software (v.4.0.1). Tumor volumes were calculated by manual slice-by-slice contouring of the gross tumor volume on contrast-enhanced CT images across all visible tumor-containing slices. All volumetric measurements were performed by the same investigator using a consistent segmentation approach to reduce interobserver variability. The volume of each segmented region of interest was recorded in cubic centimeters (cm^3^). Measurements were performed using the same software and measurement approach for all available imaging time points. Response classification followed previously published criteria [12]: CR, complete disappearance of the lesion; PR, ≥65% reduction in tumor volume; PD, ≥40% increase; and SD, volumetric changes not meeting PR or PD criteria.

RECIST-like response assessment was based on previously reported veterinary imaging response criteria. Volumetric response thresholds were adopted from published veterinary oncology studies and selected to provide volumetric equivalents to conventional response categories. These criteria were used to facilitate comparison between linear and volumetric response assessment methods while maintaining consistency with previously reported veterinary oncology literature.

Different response thresholds were applied to the linear and volumetric methods because volumetric measurements are inherently more sensitive to dimensional alterations, with the tumor volume being proportional to the cube of the linear dimensions. Therefore, smaller linear changes might correspond to larger proportional volumetric changes.

Response evaluations were performed at each available follow-up imaging timepoint. The best overall response was defined as the greatest reduction observed during the posttreatment observation period. Tumor progression was defined as imaging evidence of regrowth meeting the PD criteria using either of the measurement methods.

Follow-up Imaging Protocol

Post-treatment imaging was guided by clinical judgment, anesthetic risk, and owner consent, rather than a fixed predefined schedule. Thoracic and head CT examinations were recommended approximately 2–3 months after the completion of radiation therapy, with subsequent imaging considered when clinical signs suggested tumor progression or recurrence, when neurological signs developed, and when both owner consent and anesthetic safety permitted further evaluation. Because this study was retrospective and involved a predominantly geriatric population, follow-up intervals were not strictly standardized.

Rationale for Imaging Timing

Evaluation timing was interpreted considering the known radiobiological response patterns of sinonasal tumors. Prior clinical experience and serial imaging observations suggested that maximum tumor regression commonly occurred between 2 and 4 months post-treatment, whereas tumor regrowth was most frequently suspected between 3 and 6 months. Accordingly, images obtained within these windows were considered particularly informative for the assessment of treatment response and progression.

Definition of Radiological Progression

Radiological progression was defined separately for the linear and volumetric assessments. For the RECIST-like evaluation, PD was defined as a ≥20% increase in the sum of measured tumor diameters relative to the smallest previously recorded value, following the Veterinary Cooperative Oncology Group (VCOG) response evaluation criteria for solid tumors in dogs [11]. For volumetric assessment, PD was defined as a ≥40% increase in tumor volume compared with the minimum recorded volume, according to previously published veterinary oncology criteria [12].

To minimize the risk of misclassification due to measurement variability or imaging artifacts, progression was interpreted in conjunction with a visual assessment of tumor morphology and consistency across sequential imaging studies. When progression was suspected, confirmation was performed using follow-up contrast-enhanced CT imaging whenever available.

Handling of Incomplete Imaging

Uniform serial CT follow-up was not feasible for all cases due to anesthetic risk, owner decisions, or clinical stability. In such cases, survival and clinical outcome data were retained for analysis, whereas imaging-based comparisons were restricted to the available datasets.

Clinical vs. Imaging-Driven Follow-up

Clinical monitoring remained the principal determinant of continued management. Imaging was considered adjunctive rather than mandatory, reflecting real-world veterinary oncology practice where repeated anesthesia may impose a significant risk.

Statistical Analysis

Statistical analyses were performed using Prism software (v.9.5.1, GraphPad Software, San Diego, CA, USA). OS was calculated from the start of RT until death from any cause. Deaths from any cause were considered events for the primary OS analysis.

Survival distributions were estimated using the Kaplan–Meier method. Median survival times and corresponding 95% confidence intervals (CI) were calculated. Group comparisons were considered exploratory given the limited sample size. Formal hypothesis-driven statistical testing was therefore interpreted with caution. No formal statistical comparison between stage 4a and stage 4b groups was performed because of the very limited number of stage 4a cases (n = 4) and the resulting imbalance between groups. Descriptive statistics were used to summarize the tumor response metrics and follow-up intervals.

Survival Endpoint Definition

The primary endpoint of the study was overall survival (OS). OS was defined as the interval from the first radiation treatment to death from any cause or the last known follow-up. A prespecified sensitivity analysis was conducted to explore the potential impact of non-tumor-related mortality. In this analysis, one dog that died from an intra-abdominal hemorrhage was censored rather than treated as an event.

Time to Local Progression

Time to local progression (TTLP) was defined as the interval from the start of RT to radiological progression detected on follow-up CT. TTLP could be determined only in cases with imaging-confirmed progression and was, therefore, analyzed descriptively.

Adverse Events/Toxicity Assessment

Adverse events potentially associated with RT were identified through a review of medical records and were categorized descriptively. Attention was paid to neurological signs, ocular complications, dermatologic reactions, and acute radiation-associated effects. Ocular adverse events were retrospectively graded using VRTOG criteria based on available medical records and clinician descriptions when sufficient information was available. Owing to the retrospective design, toxicity assessment was based on clinician documentation rather than prospective predefined toxicity scoring.

Seizure activity observed after RT was recorded, and when possible, imaging findings were reviewed to evaluate potential associations between tumor progression and treatment-related effects.

## 3. Results

Case Population

In total, 20 dogs met the inclusion criteria. All dogs were classified as having modified Adams stage 4 at the time of treatment initiation. Substage classification based on imaging findings identified 4 dogs with stage 4a disease and 16 dogs with stage 4b disease. The tumor histopathology was heterogeneous and included squamous cell carcinoma, adenocarcinoma, undifferentiated carcinoma, transitional carcinoma, and sarcoma. No cases were excluded based on histopathological diagnosis. Patient characteristics, treatment details, adjunctive therapies, follow-up CT timing, and outcomes are summarized in Table 1. Adjunctive therapies were administered in nine dogs: toceranib phosphate in three, hyperthermia in four, and both toceranib phosphate and hyperthermia in two. Re-irradiation was performed in three dogs. Re-irradiation was proposed when follow-up CT demonstrated local tumor regrowth after the initial RT course. The number of additional fractions varied among cases according to clinical status, owner preference, and financial considerations. Seizure activity was observed during follow-up in some re-irradiated dogs. The dog receiving the highest cumulative radiation dose (Case 3) developed eyelid deformation with secondary mechanical corneal ulceration.

Tumor Response

Tumor response was evaluated using both the RECIST-like linear assessment and volumetric analysis. Response analysis included 21 evaluable tumor components owing to one case with dual tumor pathology. Imaging responses, radiological progression, TTLP, and adverse events for individual dogs are summarized in Table 2.

Overall Response Assessment.

Best overall responses by RECIST-like criteria were CR (n = 5), PR (n = 12), SD (n = 4), and PD (n = 0). Volumetric assessment yielded CR (n = 5), PR (n = 11), SD (n = 5), and PD (n = 0). Discordance between the linear and volumetric classifications was observed in several cases, most commonly involving RECIST-like PR with volumetric SD or RECIST-like SD with volumetric PR.

Overall Survival

**Figure 1:**
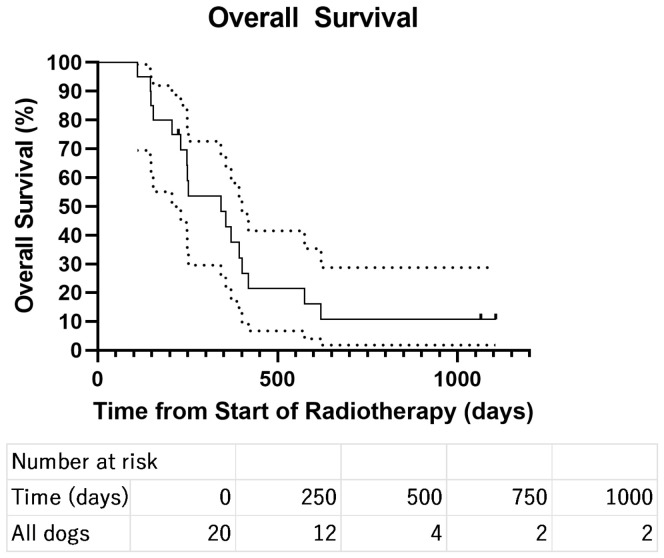
Kaplan–Meier overall survival curve for dogs with modified Adams stage 4 sinonasal tumors treated with hypofractionated radiotherapy. The median overall survival time for the entire cohort was 342 days (95% confidence interval [CI], 206–419 days). Three dogs were alive at the time of analysis and were censored at the last follow-up. Numbers at risk are shown below the survival curve.

At the time of analysis, three dogs were alive and were censored at the time of the last follow-up. The median OS for the entire cohort was 342 days (95% CI, 206–419 days), when all deaths were included as events. A sensitivity analysis was performed in which one dog that died from an intra-abdominal hemorrhage unrelated to the primary tumor was treated as a censored observation. In this analysis, the median OS was 356 days (95% CI, 231–419 days). Kaplan–Meier survival curves are shown in Figure 1.

Survival by Substage

**Figure 2:**
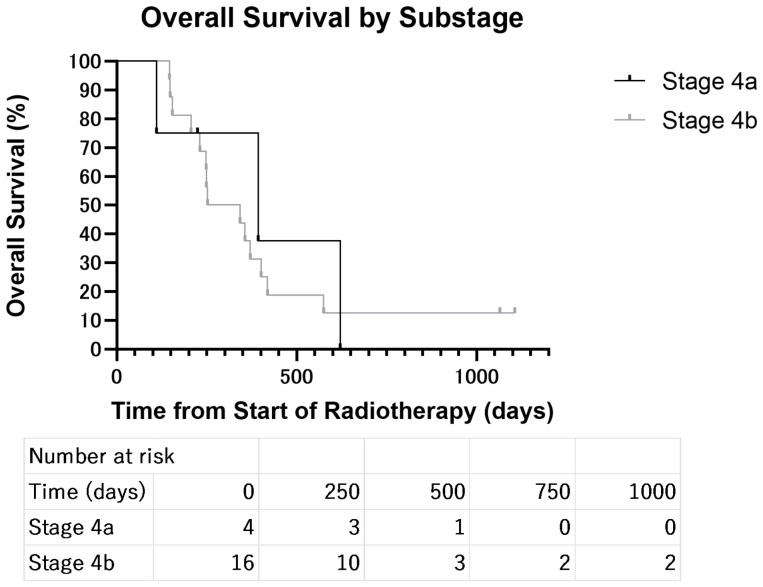
Kaplan–Meier survival curves according to tumor substage. Dogs with modified Adams stage 4a tumors (n = 4) had a median overall survival of 393 days (95% CI, 110 days to not estimable), whereas dogs with stage 4b tumors (n = 16) had a median overall survival of 297 days (95% CI, 206–401 days). Numbers at risk are shown below the survival curves.

Survival analysis based on tumor substage demonstrated differences between stages 4a and 4b. Dogs classified as having stage 4a disease had a median OS of 393 days (95% CI, 110 days—not estimable), whereas dogs classified as having stage 4b disease had a median OS of 297 days (95% CI, 206–401 days). Kaplan–Meier survival curves comparing stages 4a and 4b are shown in Figure 2. Due to the limited number of stage 4a cases (n = 4), these comparisons were considered exploratory.

Time to Local Progression

Radiological progression was confirmed on follow-up CT in eight dogs. Among these dogs, TTLP ranged from 112 to 225 days. In dogs without radiologically confirmed progression, TTLP was not evaluable because follow-up CT imaging was unavailable, insufficient, or showed no progression during the available observation period.

Patterns of Tumor Regression and Regrowth

**Figure 3:**
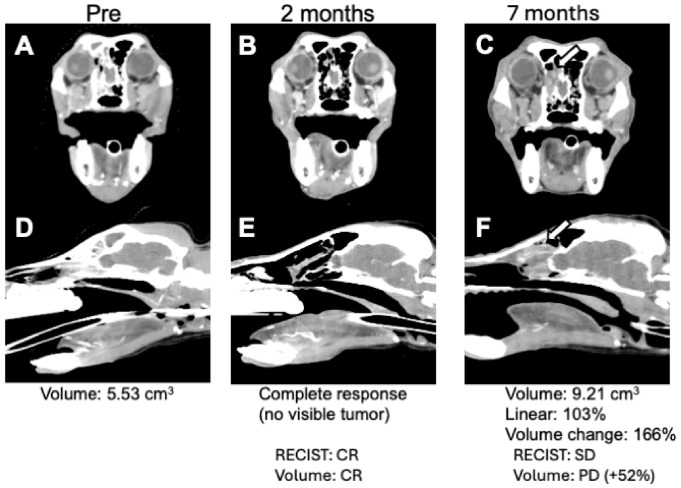
Representative contrast-enhanced CT images illustrating differences between RECIST-like and volumetric response assessment. Baseline images (**A**,**D**), complete response at 2 months after radiotherapy (**B**,**E**), and tumor regrowth at 7 months after radiotherapy (**C**,**F**; arrows) are shown. Transverse (**A**–**C**) and sagittal (**D**–**F**) planes are presented at each time point. Although complete radiological response was observed at 2 months, tumor regrowth became evident at 7 months. In this case, volumetric assessment classified the lesion as progressive disease, whereas RECIST-like assessment classified it as stable disease. This case illustrates how volumetric analysis may detect clinically relevant tumor regrowth that is less apparent using linear measurements alone.

Serial CT revealed heterogeneous patterns of tumor regression following RT. In several dogs, marked tumor reduction was observed within the first 2–4 months after treatment. Some tumors showed complete radiological disappearance during this period. However, regrowth was observed in certain cases after the initial complete response. In one representative case, complete response was documented 2 months after treatment, followed by gradual tumor regrowth detected on follow-up CT imaging at 7 months. Representative serial CT images demonstrating these changes are shown in Figure 3. These observations highlight the dynamic response patterns of advanced sinonasal tumors following hypofractionated RT.

Adverse Events

Adverse events are summarized in Table 2. Dermatologic adverse events were limited to alopecia within the radiation field, which was observed in all dogs. No severe dermatologic toxicity, including skin ulceration or necrosis, was observed.

Ocular adverse events included keratoconjunctivitis classified as VRTOG grade 1, keratoconjunctivitis sicca classified as VRTOG grade 2, and corneal ulceration classified as VRTOG grade 3. One dog developed mechanical corneal ulceration associated with eyelid deformity, which was recorded separately and was not considered primarily attributable to RT. No cases of complete vision loss were documented during the follow-up period.

Seizure activity was documented in eight dogs. The suspected cause was tumor progression in four dogs, suspected treatment-related effects in two dogs, and unclear in two dogs. The seizures were managed medically and were not associated with immediate mortality.

## 4. Discussion

In this retrospective case series, we evaluated clinical outcomes in dogs with modified Adams stage 4 intranasal tumors treated with once-weekly hypofractionated IMRT combined with SQAP. Despite the predominance of stage 4b disease, the median overall survival ranged from 342 to 356 days, depending on the censoring strategy. These outcomes are broadly comparable to previously reported survival times for dogs with advanced or modified Adams stage 4 sinonasal tumors treated with fractionated or moderately hypofractionated radiotherapy protocols [3,13]. Importantly, treatment was delivered with an acceptable toxicity profile.

Hypofractionated protocols are frequently considered palliative in veterinary oncology. However, median survival times of approximately 6–12 months after hypofractionated RT have been reported for canine nasal tumors [6,7]. The survival outcomes observed in the present cohort are therefore broadly comparable to previously reported results despite the predominance of stage 4 disease. These findings suggest that once-weekly hypofractionated IMRT may represent a clinically meaningful treatment option for selected dogs with advanced intranasal tumors. However, because no RT-alone control cohort was included and several patients received adjunctive therapies or re-irradiation, the independent contribution of SQAP to the observed outcomes cannot be determined from the present study.

Although median OS was numerically longer in dogs with stage 4a disease than in those with stage 4b disease, this comparison was exploratory because of the small and imbalanced subgroup sizes. Therefore, no definitive conclusion can be drawn regarding the prognostic impact of substage classification in the present cohort. Further investigations with larger sample sizes are warranted to clarify the prognostic impact of substage classification using hypofractionated protocols.

Parallel evaluations using RECIST-like linear measurements and volumetric analyses revealed complementary characteristics. In the present study, volumetric assessment was not intended to establish the independent antitumor effect of SQAP, but rather to characterize post-treatment tumor dynamics and explore the utility of different imaging-based response assessment methods. Discordance between RECIST-like and volumetric response classifications was observed in several cases, with volumetric assessment appearing more sensitive for detecting subtle tumor regrowth [12]. This divergence likely reflects the irregular geometric patterns of tumor regression in sinonasal structures. Volumetric methods may capture complex morphological changes more precisely, whereas linear metrics may better reflect clinically meaningful structural collapse. Although volumetric assessment requires additional time and manual contouring compared with conventional RECIST-like measurements, it may provide a more sensitive evaluation of treatment response in anatomically complex tumors such as canine intranasal neoplasms. Early detection of tumor regrowth may facilitate clinical decision-making regarding additional imaging, re-irradiation, or other therapeutic interventions. Therefore, volumetric assessment may be particularly valuable in selected cases where subtle changes in tumor burden are not adequately captured by linear measurements alone.

These findings suggest that combined interpretation of both approaches may enhance radiological assessment in advanced sinonasal tumors. Although TTLP data were limited by inconsistent imaging schedules, volumetric assessment appeared to detect progression earlier in selected cases. This observation supports the potential utility of volumetric monitoring in follow-up protocols, particularly when early detection of regrowth may influence clinical decision-making, including consideration of reirradiation or adjunctive therapies. Accordingly, in the present study, imaging-based response assessment should be interpreted as a complementary descriptive endpoint rather than as direct evidence of the independent therapeutic effect of SQAP.

In a subset of cases, adjunctive therapies including toceranib phosphate and hyperthermia were administered in addition to RT. Toceranib is a multitarget tyrosine kinase inhibitor that suppresses several signaling pathways involved in tumor angiogenesis and proliferation, including pathways mediated by vascular endothelial growth factor receptor (VEGFR) and platelet-derived growth factor receptor (PDGFR). Previous studies have reported potential clinical activity of toceranib in canine nasal carcinoma and have explored its use in combination with RT to modulate tumor vasculature and potentially enhance the radiation response [14]. Similarly, hyperthermia has been investigated as a radiosensitizing modality in both experimental and clinical oncology [15]. Thermal treatment may enhance radiation-induced cytotoxicity through mechanisms such as inhibition of DNA repair pathways, modification of tumor hypoxia, and disruption of cellular protein structures. In the present study, the number of patients receiving adjunctive therapies was limited and the treatment combinations were heterogeneous; therefore, the independent contribution of these modalities to clinical outcomes could not be evaluated.

Advanced sinonasal tumors frequently extend toward the cranial cavity and orbit, making protection of critical organs such as the brain and ocular structures a major consideration during RT planning [16]. Modern radiation techniques have been shown to reduce dose exposure to OARs while maintaining adequate target coverage [17]. In dogs with stage 4 sinonasal tumors, advanced planning approaches such as volumetric modulated arc therapy have been reported to decrease the radiation dose to the brain and optic structures compared with conventional techniques [18]. These findings highlight the importance of careful OAR management when treating tumors with intracranial extensions.

In the present study, the bilateral globes and brain were contoured as OARs during treatment planning. Despite the high proportion of stage 4b cases, severe neurological toxicity was not commonly observed, with no occurrence of complete vision loss. Although definitive conclusions regarding dose–toxicity relationships cannot be drawn from this retrospective dataset, the observed safety profile suggests that careful IMRT-based planning may allow treatment of advanced sinonasal tumors while maintaining acceptable toxicity levels.

Ocular and dermatological toxicities were observed but remained manageable. No cases of complete vision loss were observed. Severe corneal complications occurred only in a case with pre-existing orbital invasion, making attribution to radiation exposure uncertain.

Seizures were primarily documented in patients with stage 4b disease. Although seizure activity was observed in several dogs, MRI examinations were not performed at the time of onset. Therefore, it was not possible to determine whether these neurological signs were attributable to radiation-induced injury, intracranial tumor progression, or other causes. Given that most affected dogs had tumors with intracranial extension, progression of advanced local disease may have contributed to seizure development in at least some cases. However, delayed radiation-related effects cannot be excluded in dogs with prolonged survival and extended post-treatment follow-up. Importantly, despite using a hypofractionated schedule and the high proportion of tumors with intracranial extension, severe neurological toxicity was not commonly observed in this cohort. Although a longer follow-up would be required to fully characterize late radiation effects, the present findings suggest that carefully planned IMRT may mitigate the risk of excessive dose exposure to adjacent critical structures, even when hypofractionated protocols are employed. Given the advanced disease status of this cohort, the overall safety profile was considered acceptable in the context of therapeutic intent.

This study is limited by its retrospective design, small sample size, heterogeneous histopathology, and nonstandardized imaging follow-up. Additionally, adjunctive therapies were administered in nine dogs and re-irradiation was performed in three dogs, introducing treatment heterogeneity and limiting our ability to evaluate the independent contribution of SQAP, adjunctive therapies, or re-irradiation to the observed clinical outcomes. Furthermore, as no control cohort treated with RT alone was available in this retrospective dataset, the relative contribution of SQAP to treatment outcomes could not be definitively determined. Pharmacokinetic and pharmacodynamic data for SQAP in dogs remain limited; therefore, the optimal dose, timing, and therapeutic window of SQAP administration in canine patients require further investigation.

Nevertheless, our findings support the feasibility of once-weekly hypofractionated IMRT combined with radiosensitization as a treatment option for advanced intranasal tumors. Rather than replacing conventional fractionation, this protocol may represent a pragmatic alternative for cases in which the anesthetic burden, resource limitations, or patient condition restrict prolonged treatment schedules. Prospective multi-institutional studies are warranted to validate these findings.

From a clinical perspective, treatment options for dogs with advanced intranasal tumors are often limited by the need for repeated anesthesia and prolonged treatment schedules associated with conventional fractionated RT. Once-weekly hypofractionated IMRT may provide a practical alternative that maintains meaningful tumor control while reducing the treatment burden for both patients and owners. The combination of modern conformal RT techniques with radiosensitization strategies may further expand the therapeutic window for tumors involving the cribriform plate or adjacent critical structures.

## 5. Conclusions

Once-weekly hypofractionated IMRT combined with concurrent SQAP was associated with clinically meaningful survival outcomes in dogs with modified Adams stage 4 intranasal tumors. RECIST-like and volumetric response assessments provided complementary information, and volumetric analysis appeared useful for detecting tumor regrowth in selected cases. However, because of the retrospective design, treatment heterogeneity, and absence of a radiotherapy-alone control group, the independent contribution of SQAP to treatment outcomes could not be determined. Prospective controlled studies are warranted to further evaluate the role of SQAP and the clinical utility of volumetric response assessment in canine intranasal tumors.

## Figures and Tables

**Table 1:** Clinical characteristics, treatment details, and adjunctive therapies in 20 dogs.

Case	Breed	Age (Years)	Sex	Histopathology	Modified Adams Stage	RT Protocol	Adjunctive Therapy	Re-Irradiation	Follow-Up CT	OS (Days)	Outcome
1	Welsh Corgi	12.7	M	SCC	4b	8 Gy × 4	None	Yes (8 Gy × 3)	1, 2, 3, 4.5, 5.5, 6, 8 mo	252	Dead
2	Pomeranian	12.6	M	SCC	4b	8 Gy × 5	None	Yes (8 Gy × 4)	1, 2, 3, 4, 5, 7 mo	248	Dead
3	Great Pyrenees	7.7	MN	Undifferentiated carcinoma	4b	8 Gy × 5	Toceranib	Yes (8 Gy × 5)	1, 2, 4, 6, 8.5, 9.5 mo	401	Dead
4	Welsh Corgi	12.7	FS	Transitional cell carcinoma	4b	8 Gy × 6	Toceranib	No	1, 2, 6 mo	419	Dead
5	Labrador Retriever	13.8	FS	Sarcoma	4b	8 Gy × 5	None	No	1, 3 mo	148	Dead
6	Yorkshire Terrier	14.9	MN	Adenocarcinoma	4b	8 Gy × 5	None	No	1, 3, 5 mo	575	Dead
7	Welsh Corgi	7.0	FS	Adenocarcinoma	4b	8 Gy × 6	Hyperthermia	No	1, 3, 6, 14 mo	1072+	Alive
8	Miniature Dachshund	10.9	MN	Undifferentiated carcinoma	4b	8 Gy × 5	Hyperthermia	No	1, 2, 3 mo	342	Dead
9	Shiba Inu	6.5	MN	SCC	4b	8 Gy × 5	Toceranib + Hyperthermia	No	2, 6, 11, 15, 19 mo	1035+	Alive
10	Shiba Inu	12.1	MN	Carcinoma	4b	8 Gy × 5	None	No	1, 2 mo	154	Dead
11	Toy Poodle	13.4	FS	Adenocarcinoma	4b	8 Gy × 5	Toceranib	No	1, 2 mo	146	Dead
12	Shiba Inu	8.8	MN	Adenocarcinoma	4a	8 Gy × 5	None	No	2, 5, 7 mo	621	Dead
13	Mixed	11.2	MN	Carcinoma	4b	8 Gy × 5	None	No	1, 3 mo	356	Dead
14	Mixed	14.8	FS	Adenocarcinoma	4b	8 Gy × 5	Hyperthermia	No	3 mo	371	Dead
15	Shetland Sheepdog	14.3	MN	Adenocarcinoma	4a	8 Gy × 5	Hyperthermia	No	2 mo	393	Dead
16	Shiba Inu	14.0	MN	Carcinoma	4b	8 Gy × 5	Toceranib + Hyperthermia	No	3 mo	249	Dead
17	Papillon	14.3	FS	SCC or Adenocarcinoma	4b	8 Gy × 5	None	No	1.5, 2.5, 5 mo	206	Dead
18	Papillon	13.3	FS	Transitional cell carcinoma or Olfactory neuroblastoma	4b	8 Gy × 5	None	No	2.5, 4 mo	231	Dead
19	Shih Tzu	9.8	FS	Adenocarcinoma	4a	8 Gy × 5	None	No	1.5 mo	110	Dead
20	Mixed	14.8	FS	Osteosarcoma/Undifferentiated carcinoma	4a	8 Gy × 5	None	No	2 mo	312+	Alive

RT, radiation therapy; OS, overall survival; SCC, squamous cell carcinoma; M, intact male; MN, neutered male; FS, spayed female. Radiation therapy was delivered once weekly. Follow-up CT timing indicates months after completion of the initial RT course. Re-irradiation indicates additional radiation therapy performed after the initial RT course.

**Table 2:** Imaging response, radiological progression, and adverse events.

Case	Best RECIST-Like Response	Best Volumetric Response	Radiological Progression	TTLP (Days)	Ocular Adverse Events	Dermatologic Adverse Events	Seizure	Time to Seizure Onset After RT Completion	Suspected Cause of Seizure
1	PR	PR	Yes	203	Corneal ulceration, G3, ipsilateral	Alopecia only	Yes	8 mo	Tumor progression
2	PR	SD	Yes	118	Corneal ulceration, G3, ipsilateral Keratoconjunctivitis, G1, contralateral	Alopecia only	Yes	6 mo	Tumor progression
3	PR	PR	Yes	213	Mechanical corneal ulceration due to eyelid deformity, ipsilateral Keratoconjunctivitis, G1, contralateral	Alopecia only	No	NA	NA
4	CR	CR	Yes	225	Keratoconjunctivitis, G1, ipsilateral	Alopecia only	No	NA	NA
5	SD	SD	NE	NE	Keratoconjunctivitis, G1, ipsilateral	Alopecia only	No	NA	NA
6	CR	CR	No	NE	None	Alopecia only	Yes	13 mo	Suspected treatment-related effect
7	CR	CR	No	NE	KCS, G2, contralateral Keratoconjunctivitis, G1, ipsilateral	Alopecia only	Yes	14 mo	Suspected treatment-related effect
8	PR	PR	NE	NE	KCS, G2, ipsilateral	Alopecia only	Yes	3 mo	Unclear
9	CR	CR	No	NE	None	Alopecia only	No	NA	NA
10	PR	SD	Yes	112	KCS, G2, ipsilateral	Alopecia only	No	NA	NA
11	PR	PR	NE	NE	None	Alopecia only	No	NA	NA
12	CR	CR	Yes	171	None	Alopecia only	No	NA	NA
13	PR	SD	NE	NE	KCS, G2, bilateral	Alopecia only	No	NA	NA
14	PR	PR	NE	NE	None	Alopecia only	Yes	8 mo	Unclear
15	PR	PR	NE	NE	None	Alopecia only	No	NA	NA
16	PR	PR	NE	NE	Corneal ulceration, G3, ipsilateral	Alopecia only	No	NA	NA
17	PR	PR	Yes	180	Keratoconjunctivitis, G1, ipsilateral	Alopecia only	Yes	5 mo	Tumor progression
18	SD	PR	Yes	176	Keratoconjunctivitis, G1, ipsilateral	Alopecia only	Yes	4 mo	Tumor progression
19	SD	SD	NE	NE	Keratoconjunctivitis, G1, ipsilateral	Alopecia only	No	NA	NA
20	SD (Osteosarcoma)/PR (Undifferentiated carcinoma)	PR (Osteosarcoma)/PR (Undifferentiated carcinoma)	NE (both components)	NE (both components)	Keratoconjunctivitis, G1, ipsilateral	Alopecia only	No	NA	NA

CR, complete response; PR, partial response; SD, stable disease; TTLP, time to local progression; KCS, keratoconjunctivitis sicca; NA, not applicable; NE, not evaluable. Ipsilateral and contralateral refer to the side of the primary nasal tumor. NE indicates that follow-up CT imaging was unavailable or insufficient for evaluation. TTLP was calculated only in dogs with radiologically confirmed progression. Dermatologic adverse events were limited to alopecia within the radiation field in all dogs. VRTOG ocular toxicity grades were assigned retrospectively based on available medical records and clinician descriptions. Mechanical corneal ulceration associated with eyelid deformity was recorded separately and was not considered primarily attributable to RT. In case 20, responses were evaluated separately for two tumor components.

## Data Availability

The data presented in this study are available on request from the corresponding author. The data are not publicly available due to privacy and ethical restrictions related to client-owned animals.
